# BCL-2 inhibition impairs mitochondrial function and targets oral tongue squamous cell carcinoma

**DOI:** 10.1186/s40064-016-3310-2

**Published:** 2016-09-21

**Authors:** Lei Xiong, Yi Tang, Zhaoyang Liu, Jing Dai, Xiaozhou Wang

**Affiliations:** 1 Department of Oral Medicine, The Second Clinical Medical College, Yangtze University, Jingzhou Central Hospital, Jingzhou, People’s Republic of China; 2 Department of Clinical Medicine, Hubei College of Chinese Medicine, Academy Road 87, Jingzhou, 434020 People’s Republic of China

**Keywords:** Tongue squamous carcinoma, Bcl-2, ABT-199, Mitochondria

## Abstract

**Purpose:**

To understand the role of Bcl-2 overexpression in oral tongue squamous cell carcinoma (OTSCC) patients and investigate the efficacy of targeting Bcl-2 in OTSCC.

**Methods:**

The expression level of Bcl-2 on normal tongue cells and OTSCC cells were measured by real-time PCR and western blotting. The functional roles of Bcl-2 were examined by MTS, flow cytometry and xenograft cancer mouse model. Mechanism studies were performed by analyzing mitochondrial functions in a panel of OTSCC cell lines.

**Results:**

Bcl-2 is up-regulated at mRNA and protein levels in a panel of OTSCC cell lines compared to normal tongue epithelial cells (NTEC). Importantly, overexpression of Bcl-2 confers resistance of OTSCC cells to chemotherapeutic drug cisplatin treatment. Overexpression of Bcl-2 in NTEC significantly increased cell growth. In contrast, inhibition of Bcl-2 by genetic and pharmacological approaches inhibits proliferation and induces apoptosis in OTSCC cells. Mechanistically, Bcl-2 inhibitor ABT-199 impairs mitochondrial functions as shown by the decreased levels of mitochondrial membrane potential, mitochondrial respiration and ATP, and the increased levels of ROS in OTSCC cells. In addition, ABT-199 inhibits proliferation and induces apoptosis and mitochondrial dysfunctions in NTEC cells, but to a less extent than in OTSCC cells. We further show that ABT-199 augments the effects of cisplatin in eliminating OTSCC cells in in vitro tongue cancer cellular system and in vivo tongue cancer xenograft mouse model.

**Conclusions:**

Inhibition of Bcl-2 effectively targets OTSCC cells through inhibiting proliferation and inducing apoptosis. Inhibition of Bcl-2 also augments the inhibitory effects of cisplatin in vitro and in vivo.

**Electronic supplementary material:**

The online version of this article (doi:10.1186/s40064-016-3310-2) contains supplementary material, which is available to authorized users.

## Background

The clinical management of the majority of the oral cancer patients are still challenge with the 5-year overall survival and disease-free survival remaining ~55 and ~60 % (Goldstein et al. 2013). Oral tongue squamous cell carcinoma (OTSCC) is a subtype of oral cancer, which is more clinically aggressive with rapid local invasion and a high recurrence rate (Tan et al. 2012). OTSCC has increased incidence over the last several years and poor prognosis (Garnaes et al. 2015). Current treatment for OTSCC include surgery (using microvascular reconstructive techniques), radiotherapy (e.g. external beam radiotherapy and brachytherapy), chemotherapy (e.g. cisplatin) and various combinations of these modalities depending on the disease stages and presentations (Ferlay et al. 2015; Shiboski et al. 2005; Huang and O’Sullivan 2013; Andreadis et al. 2003). The molecular pathogenesis of OTSCC and its underlying mechanisms to chemotherapy resistance are not well understood. Research have found that epigenetic and genetic factors, such as oncogenes (e.g. Ras) and tumor suppressor genes (e.g. p53), can significantly influence the development of OTSCC (Khan and Bisen 2013; Murugan et al. 2012). It is therefore important to elucidate the mechanisms involved in the resistance and identify effective targets for patients with OTSCC.

Activation of pro-survival B cell lymphoma 2 (BCL2) family genes (e.g. MCL1, BCL2 and BCLX) is common hallmark of cancer and contributes to tumorigenesis via BCL2-mediated apoptosis (Adams and Cory 2007). BCL-2 is transcriptionally up-regulated in response to cytokines or pathways involved in proliferation, such as PI3K/AKT and Ras (Kinoshita et al. 1995; Franke et al. 2003). Downregulation of Bcl-2 by using ABT-199, a potent and selective inhibitor of Bcl-2 (Souers et al. 2013), has been shown to inhibit growth of a panel of cancers (Ko et al. 2014; Goff et al. 2013). Several studies have been shown that the expression of Bcl-2 family proteins are associated with clinical stage, histologic grade and poor prognosis in OTSCC patients (Camisasca et al. 2009; de Vicente et al. 2006; Zhang et al. 2012). However, little is known about the functional roles of Bcl-2 in OTSCC.

In this study, we investigated the expression and roles of Bcl-2 in normal tongue cells and multiple OTSCC cell lines. Our results show that Bcl-2 is up-regulated in OTSCC compared to normal tongue cells. The up-regulation of Bcl-2 contributes to the resistance of OTSCC to chemotherapeutic drug treatment. We further show the essential roles of Bcl-2 in growth and survival of OTSCC cells. In addition, Bcl-2 inhibition effectively inhibits proliferation and induces apoptosis of OTSCC via impairing mitochondrial functions. Finally, we demonstrate that the combination of BCL-2 inhibitor ABT-199 and chemotherapeutic drug cisplatin are more effective than single drug alone in targeting OTSCC both in vitro and in vivo.

## Methods

### Cell culture and drugs

Human primary cultured normal tongue epithelial cells (NTEC, a kind gift from Dr. Sun’s laboratory and Dr. Zeng’s laboratory) (Wen et al. 2014; Song et al. 2006) were maintained in keratinocyte/serum-free medium (Invitrogen Life Technologies, US). Oral tongue squamous cell carcinoma (OTSCC) cell lines SCC-9 and SCC-25 were purchased from the American Type Culture Collection. Tca8113 and CAL27 were purchased from the Committee of the Type Culture Collection of the Chinese Academy of Sciences. Cells were cultured in RPMI 1640 medium supplemented with 10 % fetal bovine serum (HyClone, UK). ABT-199 (Catalogue No. CT-A199) and cisplatin (Catalogue No. 479306) were purchased from ChemieTek and Sigma, respectively.

### Plasmid and siRNA transfection

For Bcl-2 overexpression, OTSCC cell lines were transfected with 1.5 µg pEMD or pEMD-Bcl2 plasmids (a kind gift from Clark Distelhorst) as previously described (Wang et al. 2001). BCL-2 knockdown were carried out in TSCC lines by transfecting with 100 nM scramble siRNA or human BCL-2-specific siRNAs using Dharmafect Transfection Reagent (Dharmacon RNAi Technologies) according to manufacture’s instructions. The target sequences of human BCL-2-specific are siRNA BCL-2: AAC ATC GCC CTG TGG ATG ACT.

### Western blotting

Cellular protein were extracted by RIPA lysis buffer (50 mM Tris HCl, pH 7.4, 150 mM NaCl, 1 mM EDTA, 1 % Nonidet P-40, 1 mM Na3VO4, 1 mM NaF, and 1× protease inhibitor, Life Technologies Inc, US). Total protein content was measured using the bicinchoninic acid protein assay kit (Thermo Scientific, US). Equal amount of proteins were resolved using denaturing sodium dodecyl sulphate–polyacrylamide gel electrophoresis and analysed by western blot using antibodies against BCL-2 and β-catenin (Cell Signalling Technologies, US).

### Real-time PCR analysis

The total RNA of NTEC, SCC-25, SCC-9, CAL27 and Tca8113 cells were isolated by using TRIzol Reagent (Life technologies, CA, US). The cDNA was amplified using a SsoFast EvaGreen Supermix kit (Bio-rad. CA) and real time PCR analysis was then performed using CFX96 RT PCR system (Bio-rad, CA). The primers are for human BCL-2 (5′-CTG CAC CTG ACG CCC TTC ACC-3′ and 5′-CAC ATG ACC CCA CCG AAC TCA AAG A-3′) and β-actin (5′-AAG GAT TCC TAT GTG GGC GAC G-3′ and 5′-GCC TGG ATA GCA ACG TAC ATG G-3′).

### Measurement of proliferation and apoptosis

Cells were treated with ABT-199, cisplatin or combination for 3 days. Proliferation activity was measured by the CellTiter 96R AQueous One Solution Cell Proliferation assay kit (Promega, US). To monitor cell density, 1 × 10^5^ cells were seeded on Day 1. The cell density were counted daily for 4 days under microscope. We determined the cell density by using Trypan Blue staining and then hemocytometer. Briefly, we take the average cell count from each of the sets of 16 corner squares and multiply by 10,000; then multiply by 2 to correct for the 1:2 dilution from the Trypan Blue addition.

Apoptotic cells were stained with Annexin V-FITC (Beckman Coulter, France) and analysed on a Beckman Coulter. The percentage of Annexin V-positive cells was determined by using CXP software.

### Determination of mitochondrial membrane potential, oxygen consumption rate (OCR), reactive oxygen species (ROS) and cellular ATP level

Cells were treated with ABT-199 for 24 h. For mitochondrial membrane potential measurement, the cells were incubated with 5,5′,6,6′-tetrachloro-1,1′,3,3′-tetraethyl benzimidazolylcarbocyanine iodide (JC-1, Invitrogen) for 30 min prior to PBS washing. Labelled cells were resuspended in 500 µL PBS and flow cytometry was conducted on a Beckman Coulter FC500. The mitochondrial membrane potential level was analysed using FlowJo version 7.7.1 (TreeStar, Ashland, OR). OCR was measured using a Seahorse XF24 extracellular flux analyser (Seahorse Bioscience, US) as per manufacturer’s instructions (Varum et al. 2011). Cells in 24-well-plate were equilibrated to the un-buffered medium in a CO_2_-free incubator and then transferred to the Seahorse XF24 extracellular flux analyser for OCR measurement. To measure ROS levels, cells were stained with redox-sensitive probe Carboxy-H2DCFDA in PBS buffer. Labelled cells were re-suspended in PI buffer and the level of Carboxy-H2DCFDA was measured using Beckman Coulter FC500. ATP levels were measured by CellTiter-Glo Luminescent Cell Viability Assay (Promega, WI, US) according to the manufacturer’s instructions.

### Tongue cancer xenograft in SCID mouse

SCID mice were purchased from Animal Resources Centre Australia. All of the animal experiments were approved by the Institutional Animal Care and Use Committee of Yangtze University. The SCID mice were inoculated with 1 million SCC-9 cells subcutaneously in the flank. The inoculation volume (0.2 ml) comprised a 50:50 mixture of cells in growth media and Matrigel (BD Biosciences). Tumour length and width were measured every 3 days and tumor volume was estimated by applying the following equation: volume = length × width^2^/2. When tumors reached approximately 200 mm^3^, the mice were treated with vehicle control, oral cisplatin at 20 mg/kg, oral ABT-199 20 mg/kg or combination of both for 30 days. Tumor frozen section slides were fixed with 4 % paraformaldehyde (Sigma, US). The nuclei and cytoplasm were counterstained with hematoxylin and eosin (H&E). The tumor sections were observed under a confocal microscopy (Zeiss LSM 510, Germany).

### Statistical analyses

All data are expressed as mean ± SD. A non-parametric Student’s t test was performed using the two-tailed distribution. A p value <0.05 was considered statistically significant.

## Results

### BCL-2 is activated and confers resistance to chemotherapeutic drug treatment in tongue squamous carcinoma cells

**Fig. 1:**
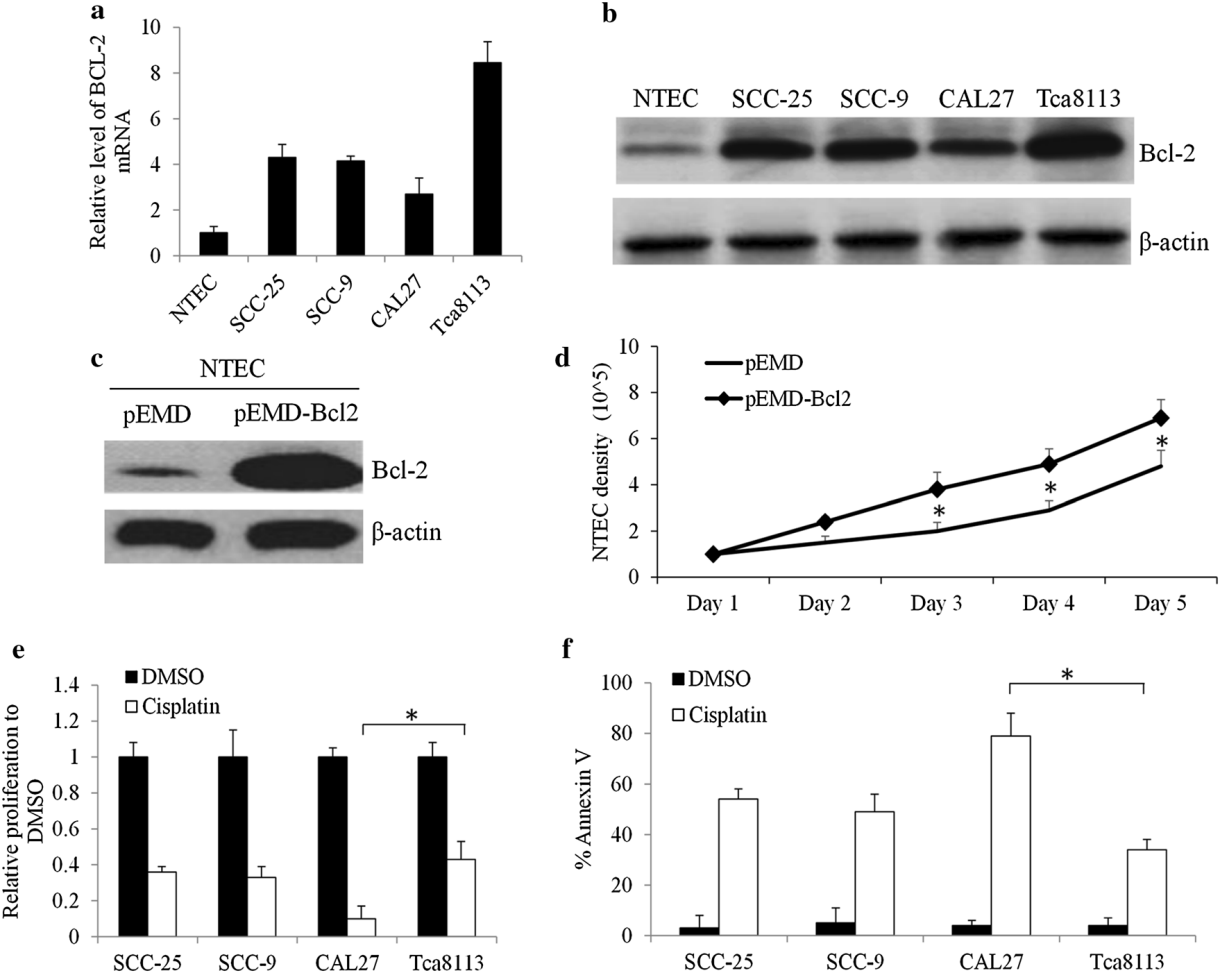
BCL-2 is up-regulated and confers resistance to cisplatin in OTSCC cells. **a** mRNA and **b** protein levels of BCL-2 in normal tongue epithelial cell (NTEC) and OTSCC cell lines. **c** Protein levels of Bcl-2 after Bcl-2 transfection (pEMD-Bcl2) in NTEC cells. **d** Overexpression of Bcl-2 promotes NTEC growth. Cells are electroporated with 1.5 μg pEMD, or pEMD-Bcl2 and cultured for 48 h prior to WB and monitoring growth. Different inhibitory effects of cisplatin on the proliferation (**e**) and survival (**f**) in SCC-25, SCC-9, CAL27 and Tca3188 cells. Cisplatin at 5 µM was used. Data were presented as mean ± SD. *p < 0.05

To understand the roles of BCL-2 in OTSCC cells, we investigated the expression levels of BCL-2 in multiple OTSCC cell lines and normal tongue epithelial cells (NTEC). Consistent with the previous studies de Vicente et al. (2006) and Mallick et al. (2009), we found that both mRNA and protein expression levels of BCL-2 were significantly increased in all tested OTSCC cell lines compared to NTEC (Fig. 1a, b; Additional file 1: Figure S1). Among four OTSCC cell lines, BCL-2 expression is highest in Tca8113 and lowest in CAL27. Compared to parental NTEC cells, we further found that there is a significant increase in proliferation in NTEC cells with BCL-2 overexpression (Fig. 1c, d), suggesting the role of BCL-2 in cell growth. Notably, chemotherapeutic drug cisplatin at 5 µM is less effectively in inhibiting proliferation and inducing apoptosis in OTSCC cell lines with high Bcl-2 expression (e.g. Tca8113) than OTSCC cell lines with low Bcl-2 expression (e.g. CAL27) (Fig. 1e, f). These data demonstrates that Bcl-2 activation confers resistance to chemotherapy drug treatment in OTSCC.

### BCL-2 inhibition by genetic and pharmacological approaches inhibits proliferation and induces apoptosis of OTSCC cells

**Fig. 2:**
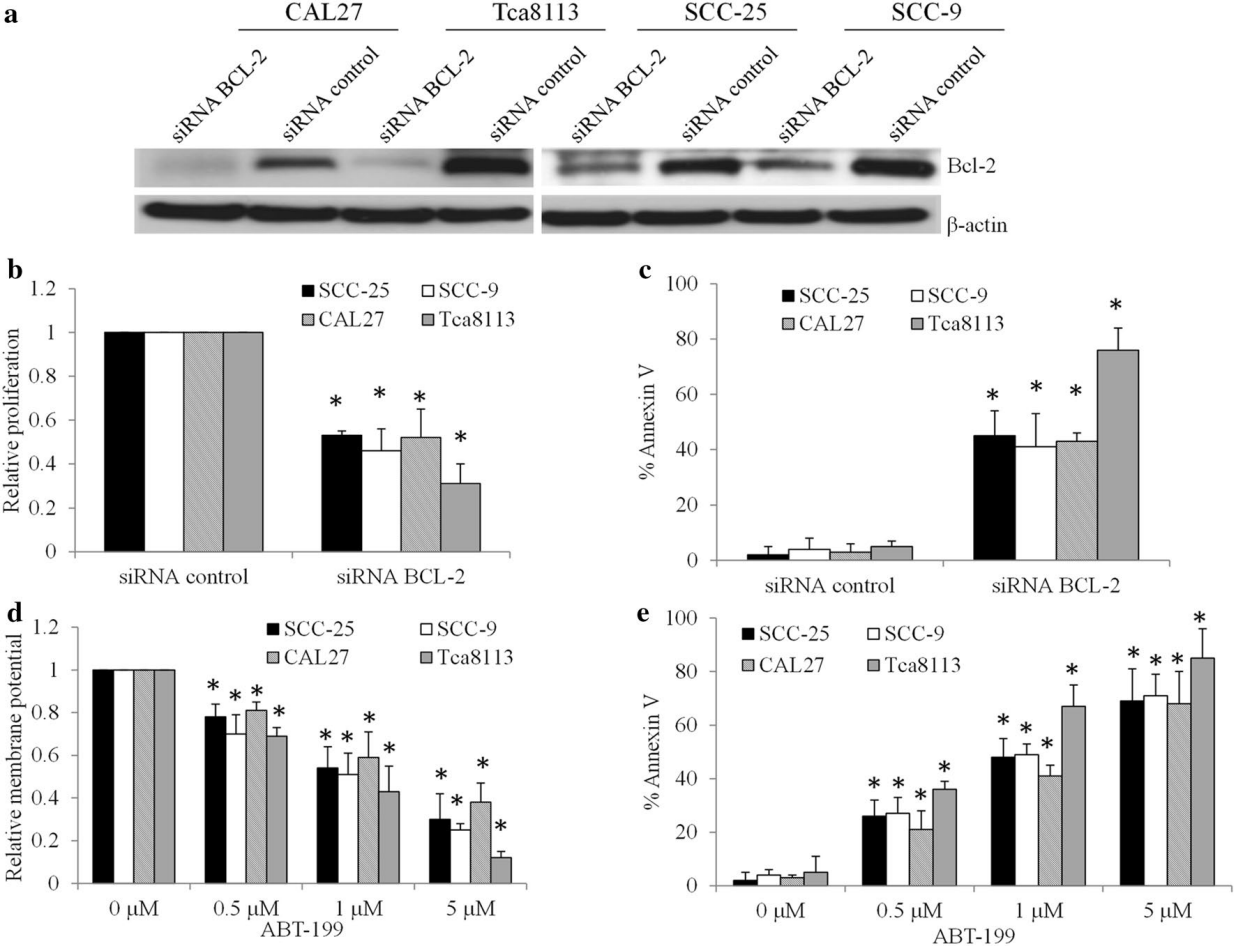
BCL-2 inhibition inhibits proliferation and induces apoptosis of OTSCC cells. **a** Bcl-2 protein expression level on BCL-2 knockdown by siRNA in SCC-25, SCC-9, CAL27 and Tca3188 cells. **b** Proliferation is decreased and **c** apoptosis is increased in Bcl-2 depleted OTSCCs. Cells are electroporated with 100 nM siRNA control or siRNA BCL-2 and cultured for 48 h prior to WB, proliferation and apoptosis assays. ABT-199 inhibits proliferation (**d**) and induces apoptosis (**e**) of OTSCCs in a dose-dependent manner. Data were presented as mean ± SD. *p < 0.05

**Fig. 3:**
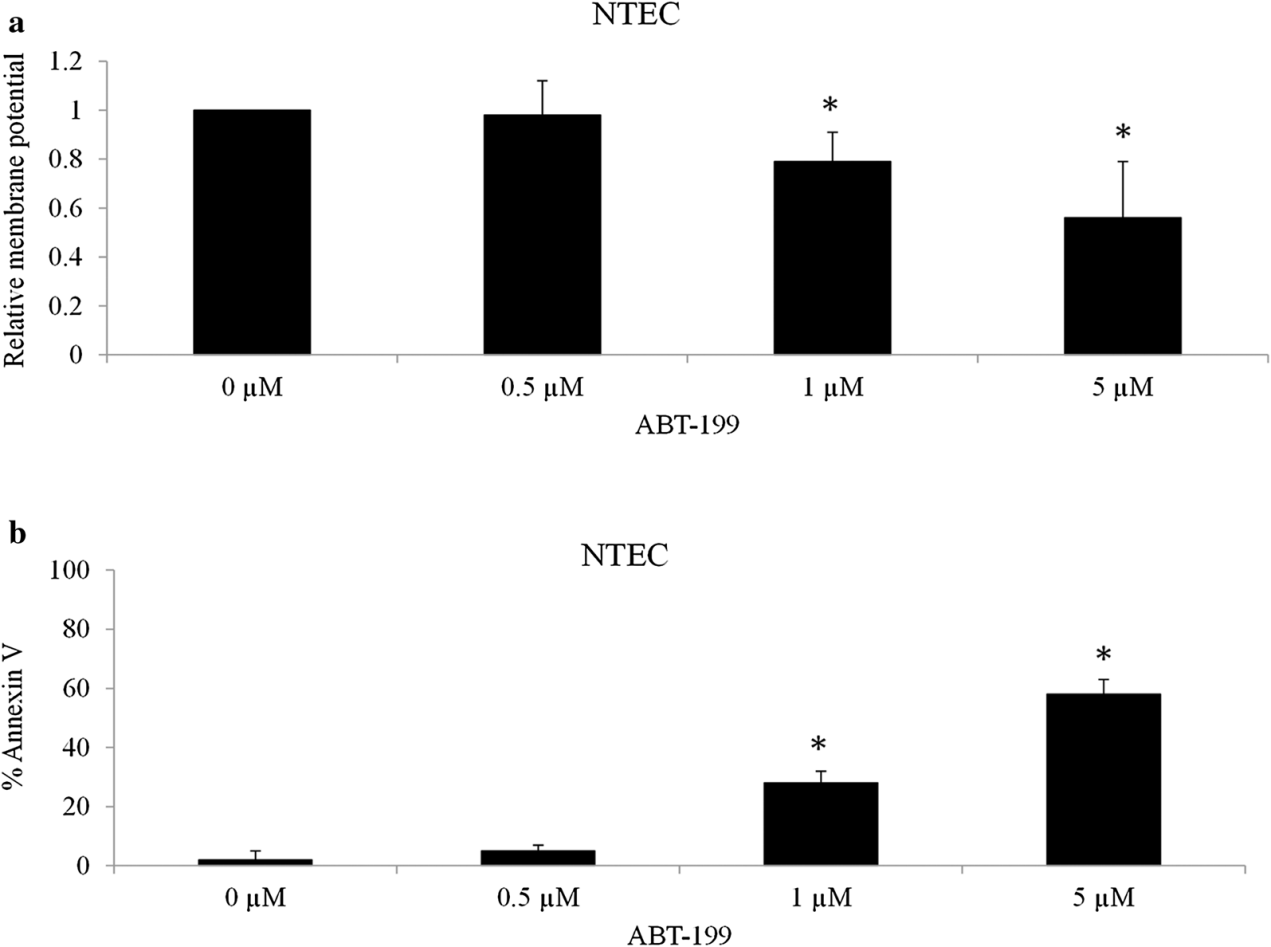
The effects of BCL-2 inhibitor ABT-199 on NTEC cells. ABT-199 at 1 and 5 µM significantly inhibits proliferation (**a**) and induces apoptosis (**b**) of NTEC cells. ABT-199 at 0.5 µM has no significant inhibitory effects in NTEC cells. Data were presented as mean ± SD. *p < 0.05

To further understand the role of Bcl-2 in OTSCC cells, we depleted BCL-2 in several OTSCC cell lines with BCL-2-specific siRNA and investigated their growth and survival. Western blot analysis showed that Bcl-2 expression levels were significantly decreased by BCL-2 siRNA but not scrambled control siRNA (Fig. 2a; Additional file 1: Figure S2). We observed that depletion of BCL-2 led to decreased growth and increased apoptosis compared to control cells (Fig. 2b, c). Consistently, we treated OTSCC cell lines with 0.5, 1 and 5 µM of ABT-199, a selective and potent BCL-2 inhibitor (Souers et al. 2013) and observed that ABT-199 dose-dependently inhibited proliferation and induced apoptosis of OTSCC cell lines (Fig. 2d, e; Additional file 1: Figure S3). In addition, it seems that Tca8113 cells are more sensitive to BCL-2 inhibition than CAL27 cells (Fig. 2b–e). We further found that ABT-199 at 1 and 5 but not 0.5 µM significantly inhibits proliferation and induces apoptosis of NTEC cells (Fig. 3), suggesting that ABT-199 targets NTEC cells to a less extent than OTSCC cells. Taken together, these data demonstrate that BCL-2 is important for OTSCC cell growth and survival.

### BCL-2 inhibition impairs mitochondrial functions in OTSCCs

**Fig. 4:**
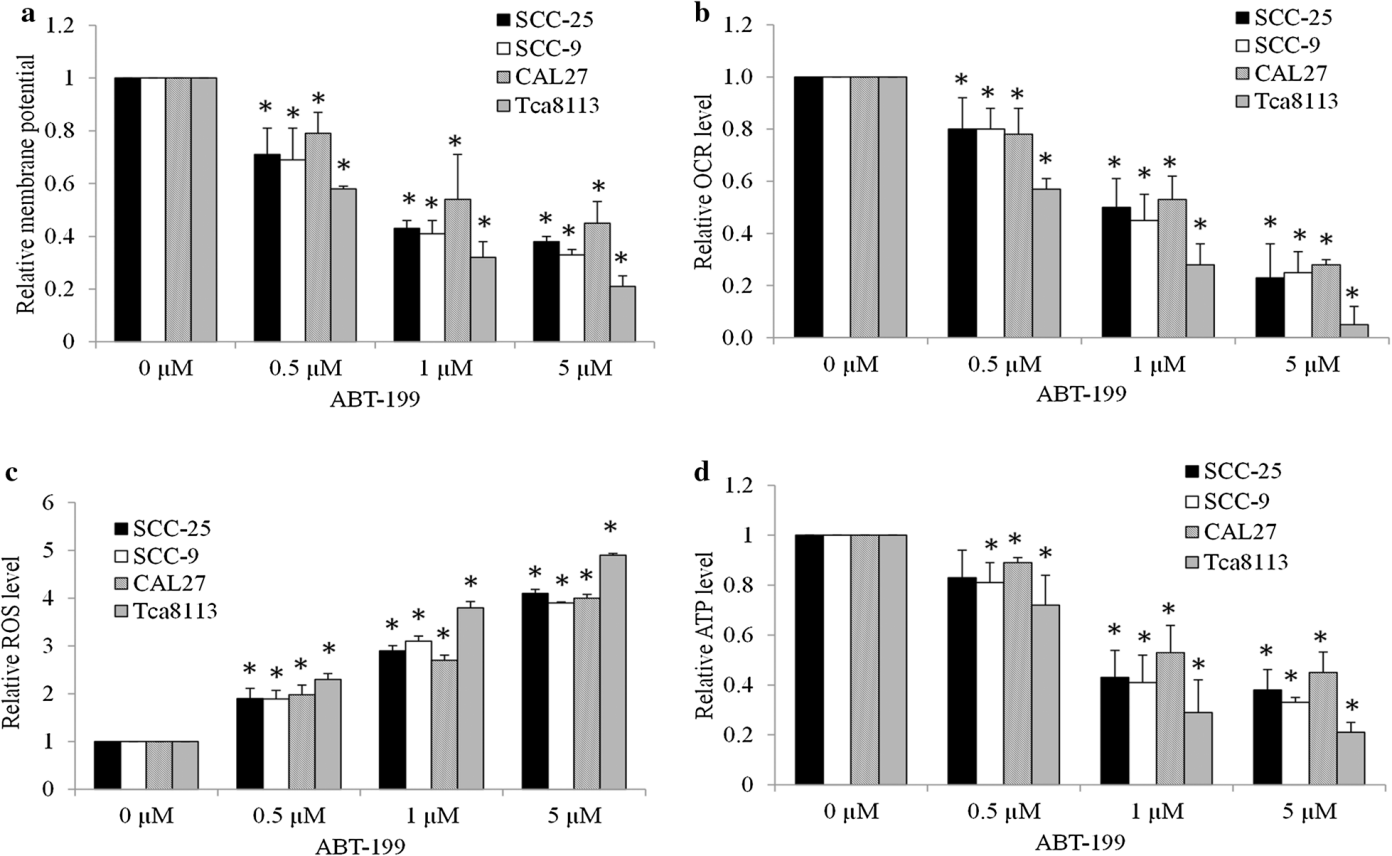
Bcl-2 inhibitor impairs mitochondrial functions in OTSCCs. **a** ABT-199 decreases mitochondrial membrane potential in SCC-25, SCC-9, CAL27 and Tca3188 cells. Cells were stained with JC-1 dye prior to flow cytometry. **b** ABT-199 dose-dependently inhibits mitochondrial respiration. OCR was measured using a Seahorse XF24 extracellular flux analyser in the absence of compound injection. ABT-199 increases ROS (**c**) and decreases ATP (**d**) levels in OTSCCs. Cells were treated with ABT-199 for 24 h prior to mitochondrial function assays. Data were presented as mean ± SD. *p < 0.05

**Fig. 5:**
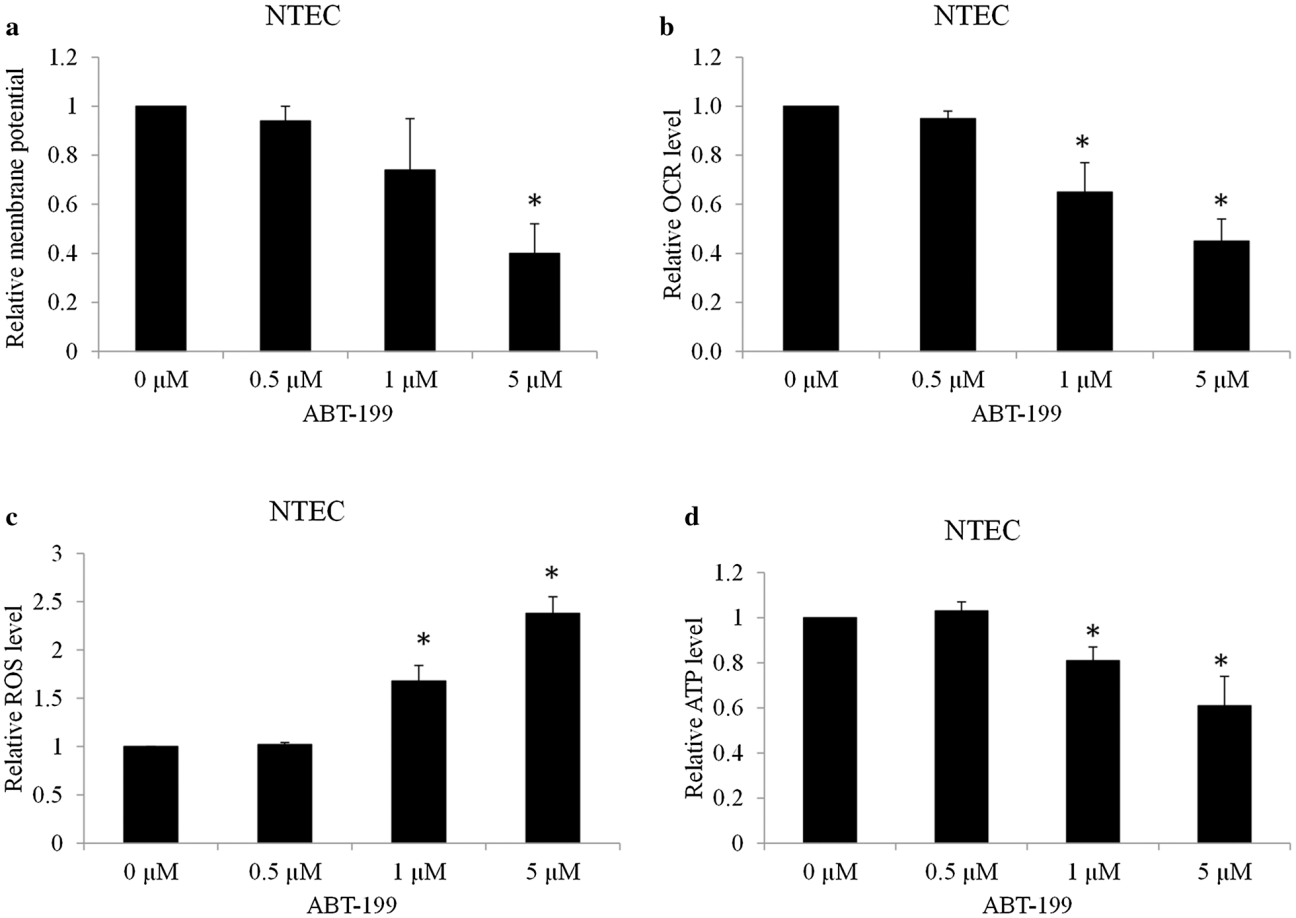
Bcl-2 inhibitor impairs mitochondrial functions in NTEC cells. ABT-199 at 1 and 5 µM decreases mitochondrial membrane potential (**a**), mitochondrial respiration (**b**), increases ROS (**c**) and decreases ATP (**d**) levels in NTEC cells. Data were presented as mean ± SD. *p < 0.05

 As Bcl-2 resides upstream of irreversible cellular damage and plays a vital role in mitochondrial functions (Gross et al. 1999), we next examined the mitochondrial membrane potential, oxygen consumption rate, levels of ROS and ATP in OTSCC cells exposed to ABT-199. We found that ABT-199 decreased mitochondrial membrane potential and mitochondrial respiration (as indicated by OCR) in a dose-dependent manner in multiple OTSCC cell lines (Fig. 4a, b). Consistent with decreased mitochondrial membrane potential and respiration, the increased ROS and decreased ATP levels were also observed in OTSCC cell lines exposed to ABT-199 (Fig. 4c, d). ABT-199 also induces mitochondrial dysfunctions in NTEC cells as shown by the decreased membrane potential, OCR, ATP levels and increased ROS levels, but to a less extent than in OTSCC cells (Fig. 5). These data show that Bcl-2 inhibition impairs mitochondrial functions in OTSCC as well as NTEC cells.

### Bcl-2 inhibitor significantly enhances the inhibitory effects of chemotherapeutic drug cisplatin in vitro and in vivo

**Fig. 6:**
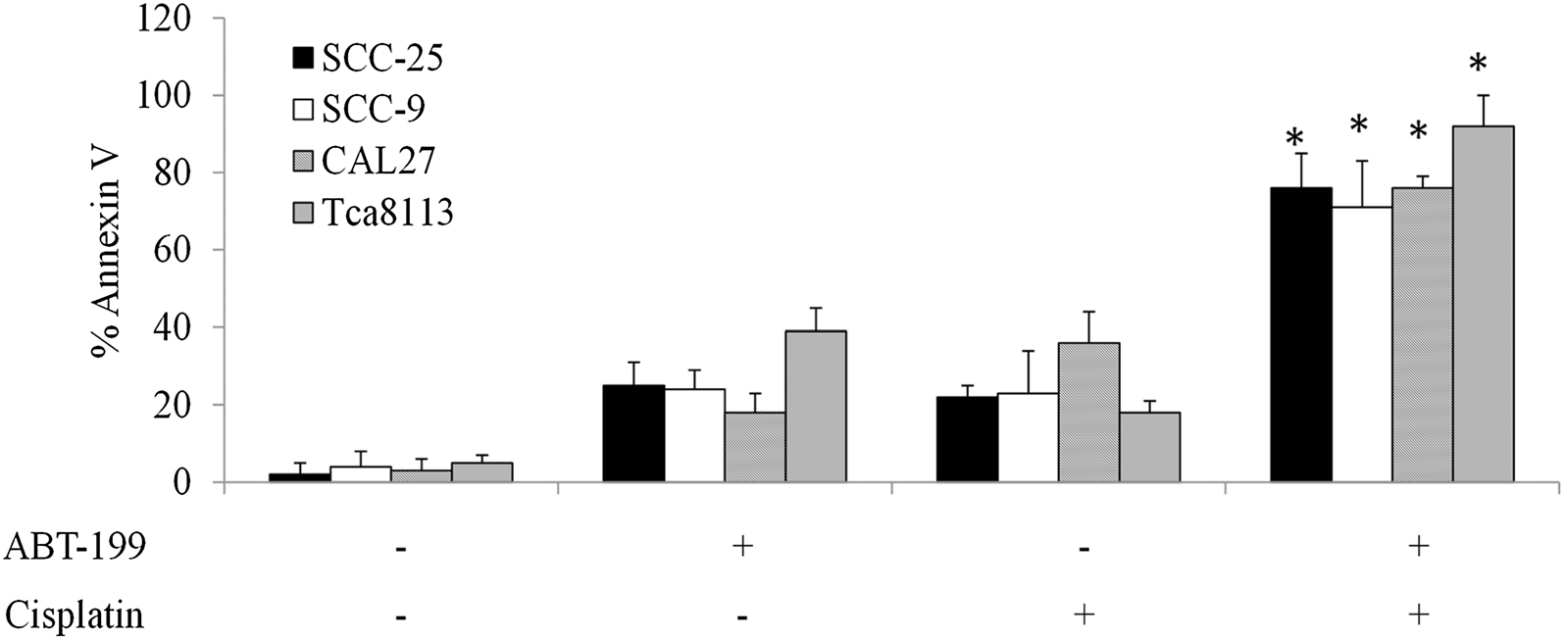
Bcl-2 inhibitor augments the inhibitory effects of cisplatin in tongue cancer cells in vitro. Combination of ABT-199 and cisplatin is superior in inducing apoptosis than single drug alone. The concentrations of ABT-199 and cisplatin used in combination studies are 0.5 and 1 µM, respectively. Data were presented as mean ± SEM. *p < 0.05, compared to single arm treatment

We have shown that Bcl-2 inhibition is active against OTSCC cells (Fig. 2). We next investigated whether its combination with tongue cancer chemotherapy drug cisplatin resulted in greater efficacy than single drug alone. We found that combination of ABT-199 (0.5 µM) and cisplatin (1 µM) induces significantly much more apoptosis than single drug alone in SCC-9, SCC-25, CAL27 and Tca8113 cells (Fig. 6).

**Fig. 7:**
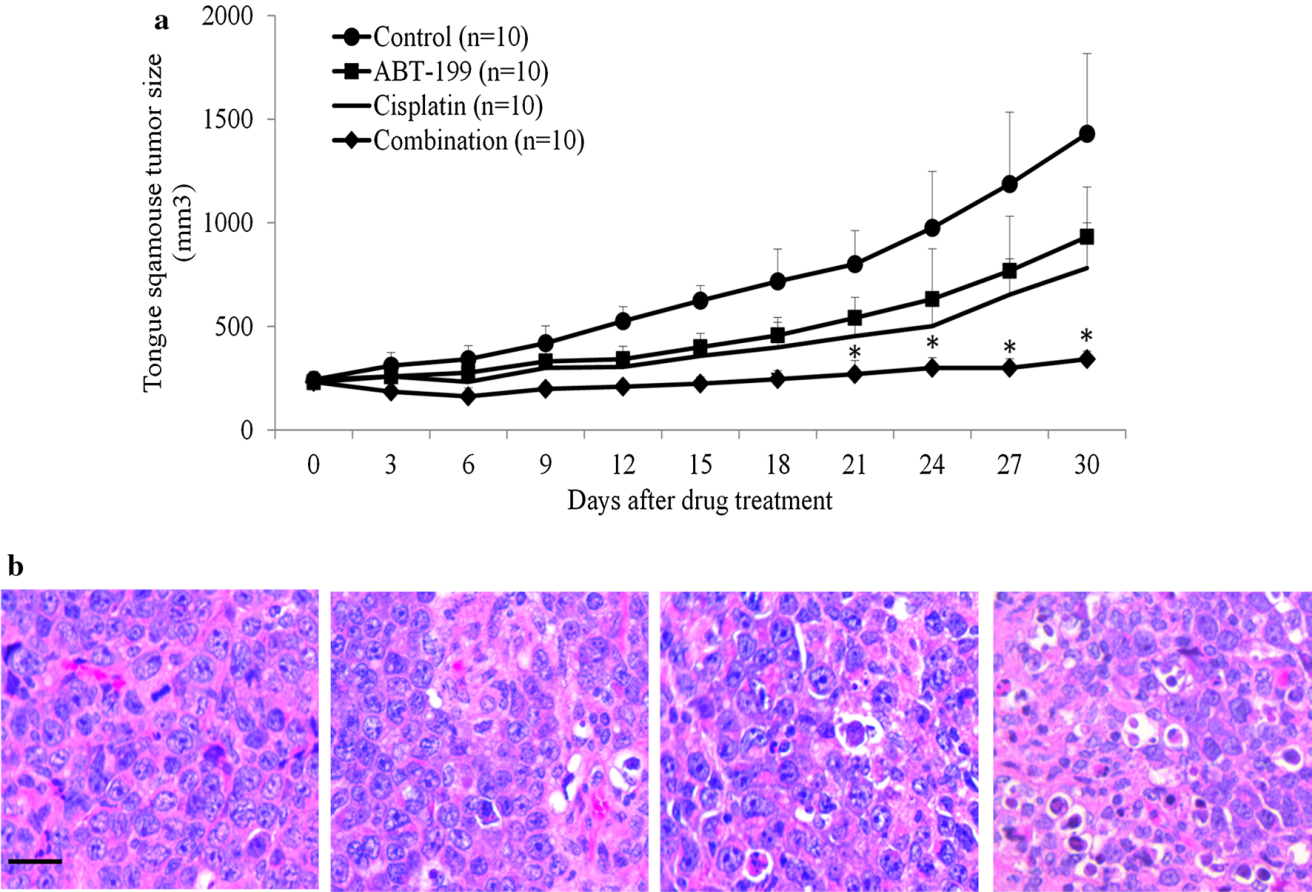
Bcl-2 inhibitor augments the inhibitory effects of cisplatin in tongue cancer cells in vivo. **a** Combination of ABT-199 with cisplatin inhibits much more growth of tongue cancer xenograft mouse model than single drug alone. ABT-199 and cisplatin inhibits growth of tumor derived from SCC-9 cells as a single agent. Combination of ABT-199 with cisplatin completely arrests tumor growth throughout the duration of treatment. There are 10 mice in each group. Data were presented as mean ± SEM. *p < 0.05, compared to single arm treatment. **b** Representative photos of H&E staining. Increased cell death shown by cytoplasmic swelling, vacuolization or disintegration were observed in combination group compared to control or single treatment group. *Scale bar* presents 50 μM

We evaluated the ability of combination to suppress tumor growth in vivo in tongue squamous tumor xenograft established in immunocompromised mice. After a single oral dose of 20 mg per kg body weight in xenograft derived from SCC-9 cells, ABT-199 and cisplatin caused a tumor growth inhibition of ~30 and ~40 %, respectively (Fig. 7a). However, when ABT-199 was combined with cisplatin, approximately 100 % tumor growth inhibition was observed throughout the duration of treatment (Fig. 7a). Consistently, H&E staining showed a marked increase in individual neoplastic cell death (indicated by cytoplasmic swelling/vacuolization/disintegration) in the combination treatment group as compared to the control and single drug treatment groups (Fig. 7b). These results were in concordance with our in vitro data, and confirmed that ABT-199 enhances the inhibitory effects of cisplatin in OTSCC.

## Discussion

OTSCC is an aggressive subtype of oral cancer and is resistant to current chemotherapy. Although several clinical studies have shown that Bcl-2 expression is associated with neck lymph node metastasis and poor prognosis in oral cancer (Camisasca et al. 2009; Mallick et al. 2009; Popovic et al. 2007), the functional roles of Bcl-2 in OTSCC and whether it is involved in the resistance of OTSCC to chemotherapy are not well understood. In this work, we are the first to demonstrate that Bcl-2 is overexpressed and plays essential roles in the growth and survival of OTSCC cells. Importantly, up-regulation of Bcl-2 confers resistance of OTSCC cells to chemotherapeutic drug treatment. In addition, Bcl-2 inhibition effectively targets OTSCC cells via impairing mitochondrial functions and augments cisplatin’s effects in eliminating OTSCC cells.

Our data that the BCL-2 mRNA and protein levels are significantly increased in multiple OTSCC cell lines compared to normal tongue epithelial cells (Fig. 1a, b) are consistent with the previous study that Bcl-2 is overexpressed in primary OTSCC tissues (Popovic et al. 2007). We further extend the previous studies by demonstrating that Bcl-2 up-regulation contributes resistance of OTSCC cells to cisplatin treatment (Fig. 1e, f) and combination of BCL-2 selective inhibitor ABT-199 and cisplatin is more effective in eliminating OTSCC cells (Fig. 6). The combination of ABT-199 and cisplatin is superior to single drug alone and completely arrests OTSCC in vivo tumor growth (Fig. 7). Taken together, our study clearly demonstrates the therapeutic value of targeting BCL-2 in overcoming chemotherapy resistance in OTSCC. Compared to other BCL-2 family inhibitors (e.g. navitoclax), ABT-199 has shown efficacy with less antiplatelet activity and a more favorable therapeutic index in patients with refractory chronic myeloid leukemia (Souers et al. 2013). In lines with the earlier publications, our work suggests that ABT-199 is a useful addition to the treatment armamentarium for OTSCC.

We further demonstrate that Bcl-2 overexpression in normal tongue epithelial cells promotes the growth (Fig. 1c, d). In contrast, knockdown BCL-2 by using siRNA or inhibition of its activity by using ABT-199 inhibits proliferation and induces apoptosis of OTSCC cells (Fig. 2). Activation of pro-survival BCL-2 has been shown to play positive roles in survival in various tumors (Adams and Cory 2007; Domen et al. 1998; Jiang and Milner 2003). In agreement with this notion, we are the first to show the protective and supportive roles of Bcl-2 on growth and survival in OTSCC.

A consequence of Bcl-2 inhibition is the mitochondrial dysfunction as demonstrated by the decreased mitochondrial membrane potential, decreased mitochondrial respiration and decreased ATP levels, and the increased ROS levels in OTSCC cells treated with selective Bcl-2 inhibitor ABT-199 (Fig. 4). This finding supports the previous study that BCL-2 inhibition targets oxidative phosphorylation in leukemia stem cells (Lagadinou et al. 2013). Besides mitochondrial respiration inhibition, our study extends the previous work by demonstrating that inhibition of Bcl-2 decreases mitochondrial membrane potential, inhibits ATP production and induces ROS generation. Targeting mitochondrial metabolism has emerged as an attractive therapeutic strategy in cancer due to their unique dependence on proper mitochondrial functions (Vander Heiden 2011; Jaras and Ebert 2011; Mayevsky 2009; Hagland et al. 2007). Our work suggests that targeting mitochondria may be an alternative therapeutic strategy in OTSCC.

In conclusion, our work on the identification of essential roles of BCL-2 in OTSCC provides better understanding of the molecular mechanisms underlying resistance of OTSCC cells to chemotherapy. Our work also suggests a rationale for targeting BCL-2 and mitochondria in OTSCC treatment.

## Additional file

10.1186/s40064-016-3310-2 Supplementary information.
