# Supplementary material for: BCL-2 inhibition impairs mitochondrial function and targets oral tongue squamous cell carcinoma
Source: Springerplus. 2016 Sep 21;5(1):1626. doi: 10.1186/s40064-016-3310-2 (PMC5031576; doi:10.1186/s40064-016-3310-2)
Supplement: Supplementary file 1 — 10.1186/s40064-016-3310-2 Supplementary information. [file 40064_2016_3310_MOESM1_ESM.doc]

**Additional file 1: Figure S1. Quantification of Bcl-2 expression levels in OTSCC cells.** Data were presented as mean ± SD. *p<0.05.

**Additional file 1: Figure S2. Quantification of Bcl-2 expression levels in OTSCC cells after siRNA transfection.** Bcl-2 protein expression level on BCL-2 knockdown by siRNA in SCC-25, SCC-9, CAL27 and Tca3188 cells. Data were presented as mean ± SD. *p<0.05.

**Additional file 1: Figure S3. The effects of Bcl-2 inhibition in OTSCC cells.** (A) Depletion of BCL-2 or (B) treatment with ABT-199 significantly induces apoptosis and necrosis of Tca8113 cells. Cells were labeled with Annexin V-FITC and PI. The percentage of Annexin V(-) PE(-) (indicates live cells), Annexin V(+) PE(-) (indicates apoptosis), Annexin V(+) PE (+) (indicates necrosis) were indicated on the FACS images.
